# Relationship between Timed Up and Go performance and quantitative biomechanical measures of balance

**DOI:** 10.3389/fresc.2024.1220427

**Published:** 2024-03-19

**Authors:** Prasad Tendolkar, Oluwaseun Ibironke, Giorgia Marchesi, Alice De Luca, Valentina Squeri, Karen J. Nolan, Rakesh Pilkar, Kiran K. Karunakaran

**Affiliations:** ^1^ Center for Mobility and Rehabilitation Engineering Research, Kessler Foundation, West Orange, NJ, United States; ^2^ Movendo Technology S.r.l., Genoa, Italy; ^3^ Department of Physical Medicine and Rehabilitation, Rutgers—New Jersey Medical School, Newark, NJ, United States; ^4^ ActiGraph, LLC, Pensacola, FL, United States

**Keywords:** traumatic brain injury, Timed Up and Go, balance, rehabilitation, biomechanical

## Abstract

Traumatic brain injury (TBI) impairs sensory–motor functions, with debilitating consequences on postural control and balance, which persist during the chronic stages of recovery. The Timed Up and Go (TUG) test is a reliable, safe, time-efficient, and one of the most widely used clinical measures to assess gait, balance, and fall risk in TBI patients and is extensively used in inpatient and outpatient settings. Although the TUG test has been used extensively due to its ease of performance and excellent reliability, limited research has been published that investigates the relationship between TUG performance and quantitative biomechanical measures of balance. The objective of this paper was to quantify the relationship between biomechanical variables of balance and the TUG scores in individuals with chronic TBI. Regression models were constructed using six biomechanical variables to predict TUG scores. The model that conservatively removed gait speed (i.e., TUG-1/GS) gave the best results, achieving a root-mean-square error of ∼±2 s and explaining over 69% of the variability.

## 1 Introduction

Traumatic brain injury (TBI) affects 2.87 million people annually in the United States and is a leading cause of long-term disability (1, 2). More than 20% of these individuals have a moderate or severe TBI with secondary motor impairments (2). TBI can impair sensory–motor functions, with debilitating consequences on balance, which persist during the chronic stages of recovery (3, 4). Individuals with TBI have reduced postural stability due to these sensory–motor deficits post-injury (5, 6). Sensory systems (vestibular, proprioceptive, and visual), sensory integration, and muscular responses show functional deficits due to TBI (3, 4). Specific to sensory systems, TBI can directly affect one or more visual, vestibular, or proprioceptive systems (3, 4). This leads to changes in how the central nervous system organizes sensory inputs for postural stability and balance, either due to deterioration in the sensory systems themselves or compensation for losses in sensory integration (5, 6). As a result, people with TBI with compromised sensory–motor functions have debilitating consequences on postural control and balance (3, 4). Failure to utilize postural stability and balance mechanisms can lead to reduced speed, control and co-ordination and an increased risk of falls in individuals post-TBI (3, 4). Thus, effective postural control is a fundamental necessity for functional mobility.

The Timed Up and Go (TUG) test is a reliable, cost-effective, safe, time-efficient, and one of the most widely used clinical methods to assess mobility, including gait, postural control, and fall risk, among individuals with TBI for both inpatient and outpatient settings (7–9). TUG assesses various motor tasks such as sit to stand, standing, walking, and turning. Thus, TUG evaluates multiple aspects of balance. TUG is known to assess activity limitations in the International Classification of Functioning (ICF), Disability, and Health model by examining the patient's ability to ambulate, balance, and perform transfers while avoiding many of the complications inherent to qualitative scoring or self-reports. TUG has shown excellent test–retest reliability (ICC = 0.86) in the TBI population and has been shown to correlate well with the Berg Balance Scale (*r* = −0.81) (7, 10). Although the TUG test has been used extensively due to its ease of performance and excellent reliability, limited research has been published investigating the relationship between TUG performance and quantitative biomechanical measures of balance. Previous research subdivided TUG into phases to understand motor function using temporal and spatial characteristics during TUG performance (11, 12). While this approach may provide insight into specific movement components that contribute to TUG performance, it did not capture the balance components or balance deficits. In fact, little focus has been placed on how functional and clinical measures might be explained through biomechanical balance parameters to predict clinical balance ability. The task of the human balance system is to keep the body's center of mass (COM) positioned over the base of support (BOS) and aligned with the center of pressure (COP) (13). The balance system constantly tries to maintain this stability, even during quiet standing, where there are small postural sways that reflect the constant activity of control (13). Perturbations, whether external or internal, disrupt the alignment between COM and COP, resulting in a loss of balance (13). Balance is maintained by using ankle or/and hip response (5, 6, 13). Posturography aims to quantify these changes to better understand balance mechanisms (13–15). COM, COP trajectory (path length, PL), and, more recently, trunk trajectory are predominantly used to quantify and understand the biomechanical characteristics of balance (13–20). Previous research used components of the Sensory Organization Test in conjunction with data from a limits-of-stability exercise to predict a questionnaire on self-perceived balance deficits (21). In this investigation, there were no significant balance-related covariate effects in the regression models (21). Thus, the objective of this paper was to quantify the relationship between biomechanical variables of balance and the TUG scores in individuals with chronic TBI. We hypothesized that TUG scores would be related to dynamic and reactive balance variables. Understanding the relationship between TUG performance and biomechanical metrics could help inform the development of personalized interventions for individuals with TBI based on their residual motor and balance deficits. In addition, the information could be used to guide recovery progression and may ultimately improve TBI rehabilitation.

## 2 Methodology

### 2.1 Participants

Twenty-one individuals diagnosed with TBI (>6 months) and presenting with unilateral or bilateral moderate-to-severe deficits at the time of injury were recruited for the study. Inclusion criteria for the participants were as follows: (1) aged between 18 and 68 years; (2) diagnosed with TBI; (3) absence of orthopedic, neuromuscular, or severe neurological pathologies unrelated to their TBI that could interfere with their ability to ambulate; and (4) ability to stand upright for 30 min with or without assistance; and cognitive capability to follow commands. One participant was excluded based on an outlier analysis performed on Timed Up and Go using MATLAB, and another participant did not complete all the balance tasks. Nineteen TBI participants were included for analysis during visit 1. Fifteen of these participants returned for visit 2. Also, 12 age-matched healthy controls (HC) were recruited for the study. Inclusion criteria for the participants were as follows: (1) aged between 18 and 68 years; (2) absence of orthopedic, neuromuscular, or neurological pathologies that could interfere with their ability to ambulate; and (3) no diagnosed cognitive disability. Demographics for all participants are presented in Table 1. All procedures performed in this investigation were approved by the Institutional Review Board at Kessler Foundation, and informed consent was obtained prior to study participation. The participants were recruited through the Kessler Foundation participant database.

**Table 1:** Participant demographics.

Participant	Sex	Age	Weight (kg)	Height (m)	TSI (years)
TBI	12 M, 7 F	41.42 ± 16.31	82.53 ± 22.22	1.71 ± 0.10	7.24 ± 8.18
HC	8 M, 4 F	46.25 ± 18.65	82.58 ± 14.65	1.74 ± 0.10	N/A

### 2.2 Data collection

Participants with TBI completed two data collection sessions spaced one month apart: visit 1 and visit 2 (occurring ∼1 month after visit 1). All participant data were collected in a dedicated gait laboratory for research purposes. This gait lab provides an unobstructed straight pathway for performing walking tasks. The data collection environment was consistent across all participants and across both visits. The objective is to quantify the relationship between the biomechanical variables of balance and the TUG scores. To validate the reliability of the computed relationship, the TBI participants repeated the tasks performed during visit 1 after ∼1 month. The participants continued their prescribed therapies to improve motor performance between visit 1 and visit 2. Healthy control participants performed one data collection session. Data collection included the following measures:

TUG: Participants walked a distance of 3 m from a seated position, turned around a cone, walked back to the chair, and sat back in the chair with their back against the backrest. The time taken to perform the task was recorded by an experienced rater (22).
Gait speed: The time to complete a 6-m walk was recorded. Participants were instructed to walk a distance of 8 m along an unobstructed pathway. The time taken to walk 6 m was recorded. The first 1 m was for acceleration and the last 1 m was for deceleration and were excluded from the speed computation. Gait speed was computed as the distance of 6 m divided by the time to complete the task (22).
Balance parameters: The balance parameters selected for this study were collected from a series of balance tasks performed on a commercially available Hunova robotic device (Movendo Technology srl, Genoa, Italy) (23). The Hunova robotic balance platform is an FDA Class II device used for objective evaluation and rehabilitation of balance and postural control in multiple populations (18, 19, 23–27). This balance measurement device includes two electromechanical platforms: one under the feet and the other under the seat (23). Each platform has a six-axis force–torque sensor that is used to estimate the antero-posterior (AP) and medio-lateral (ML) components of the COP (23). From the COP measures, built-in algorithms in the device compute the standard classical parameters, such as the resultant range of oscillation of the trunk, oscillation time of the trunk, area of the COP movement duration of the sit-to-stand task, etc. (23). Optical incremental encoders allow the measurement of the inclination of the platform (23). Simultaneously, a wireless inertial measurement unit (IMU) sensor, synchronized by Hunova software with the robotic device, was placed on the sternum of the user to monitor the trunk motion (23). IMU sensors have been reliably and widely used in previous posturography research (16–20).

**Figure 1:**
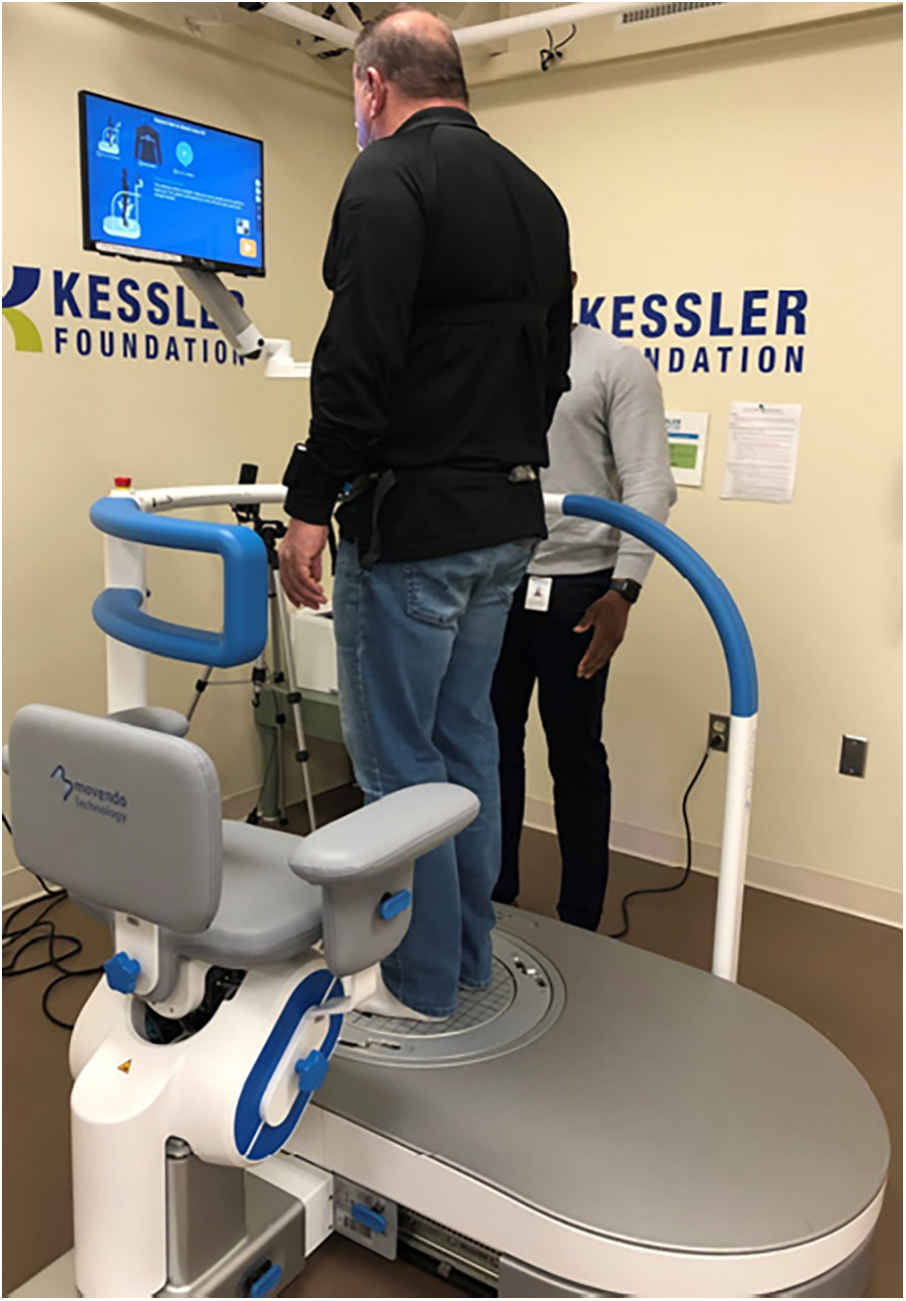
Participant diagnosed with TBI on the Hunova robotic platform.

The device enables the performance of various tasks, including exercises to understand static/dynamic/reactive balance, limits of stability, and sit-to-stand balance, as well as the measurement of COP and trunk dynamics during these tasks (18, 23, 25). The sampling rate is 100 Hz during all exercises. The following balance parameters were measured on the Hunova device for all participants (Figure 1):

(a) Static balance in “eyes open” (EO) and “eyes closed” (EC) conditions: Participants were asked to perform quiet standing and maintain their balance on the platform for 30 s. The test was performed in two conditions: with eyes open (30 s; one trial) and with eyes closed (30 s; one trial).
(b) Dynamic balance on a passive surface: Participants stood on the platform while it moved in a circular motion for 30 s (one trial). One rotation of the platform was completed in 5 s with an inclination of 3° (19). Individuals were instructed to maintain their balance while keeping their trunks as upright as possible for the duration of the test.
(c) Limits of stability: The aim of this measurement was to determine the participant's cone of stability. Participants were instructed to shift their center of gravity (COG) to reach a target that appeared on the screen in any of four possible directions and then shift their COG back to the middle (for example, in a standing position, the patient should move using his ankle range of movement, without tilting the trunk). The target distance increased if the participant reached the target. Participants shifted their weight about the ankle to reach targets shown on the screen in all three trials in each of the four directions.
(d) Reactive balance: The platform rotates randomly in three different directions (forward, left, and right) with an angular tilt at an amplitude of 6°. The foot platform moves independently from the body sway using a pre-programmed downward angular tilt along three different rotational axes. In the forward direction, the platform tilts along the *z*-direction, resulting in the toes going down. In the rightward and leftward tilt directions, the platform tilts about the *x*-axis, resulting in the right foot and left foot going down, respectively. The platform rotates following a Gaussian profile trajectory with a peak at 330 ms after the perturbation onset (mean velocity ∼16.5°/s) (18). The participants were instructed to maintain their balance and keep the trunk upright. Three perturbations were provided in each direction, and to ensure that the perturbations were unexpected, the disturbance directions and latency times were random. The first perturbation was a practice trial and was not evaluated.
(e) Sit-to-stand balance: The participants were asked to complete five trials of the sit-to-stand task. The time required to perform the task was recorded.

### 2.3 Data analysis

#### 2.3.1 Variable selection

In this exploratory cross-sectional study, we developed a linear model for the TUG test using balance parameters from a chronic TBI and a healthy control cohort to quantify the relationship between TUG performance and biomechanical parameters. The selection of variables for subsequent analysis was based on a comprehensive review of a large initial pool of 69 potential predictors from the Hunova robotic device. Variables were excluded to reduce collinearity and create a parsimonious model that could capture the essential information from all exercises. Pearson's correlation was used to select one outcome from each task to reduce co-linearity. R statistical software was used for all data analyses.

Using this guideline, the following variables were chosen:

(a) COP PL: COP path length is a widely used biomechanical metric to quantify the magnitude of the two-dimensional (AP and ML) displacement of the COP based on the distance traveled. A larger value of COP path length represents lesser postural stability (13). The robotic platform measures vertical ground reaction force in both AP and ML directions. The COP PL is the distance that the COP travels within a task, defined in Equation 1, where *n* is the total COP data points recorded (*n* is held constant for all participants). This measure was included for the static balance task.
(b) Resultant range of oscillation for static, and dynamic balance tasks: The accelerometer on the trunk is used to record the range of trunk oscillation in the antero-posterior and medio-lateral directions. We define the resultant range of oscillation as the Euclidean norm of the two range values, expressed in Equation 2.
(c) Oscillation time for reactive balance task: The robotic device records the average time taken by the participant to stabilize their trunk after each perturbation, separated by perturbation direction (forward, left, right). We took the average of these separate measures, as expressed in Equation 3.
(d) Limits of stability area: It denotes the area of the maximum COP movement in the forward/backward/right/left direction.
(e) Duration of the sit-to-stand task: The total time taken to perform five sit-to-stand tasks was recorded.
  (1) $$\;\begin{array}{rl} & \mathrm{COP}\;\mathrm{Path}\;\mathrm{Length}\\ & \;=\sum_{i=1}^{n-1}\sqrt{\begin{array}{rl} & {\left(\mathrm{CO}P_{\mathrm{ML}}(i+1)-\mathrm{CO}P_{\mathrm{ML}}(i)\right)}^{2}+({\mathrm{COP}}_{\mathrm{AP}}(i+1)-{\mathrm{CO}P_{\mathrm{AP}}(i))}^{2} \end{array}} \end{array}$$

Where *i* denotes the index of a particular COP sample, and *n* indicates the total number of samples in either COP timeseries.

(2) $$\begin{array}{rl} & \mathrm{Resultant}\;\mathrm{Range}\\ & =\sqrt{{\left(\mathrm{Trunk}\;\mathrm{Range}\;\mathrm{of}\;\mathrm{Oscillation}\;\mathrm{AP}\right)}^{2}}+{\left(\mathrm{Trunk}\;\mathrm{Range}\;\mathrm{of}\;\mathrm{Oscillation}\;\mathrm{ML}\right)}^{2} \end{array}$$

(3) $$\begin{array}{rl} & \mathrm{Reactive}\;\mathrm{Oscillation}\;\mathrm{Time}\;\mathrm{Mean}\\ & \;=\frac{\begin{array}{rl} & (\mathrm{Reactive}\;\mathrm{OT}\;\mathrm{Left}+\mathrm{Reactive}\;\mathrm{OT}\;\mathrm{Front}+\mathrm{Reactive}\;\mathrm{OT}\;\mathrm{Right}) \end{array}}{3} \end{array}$$

#### 2.3.2 Comparison between TBI participants and healthy controls

The functional and biomechanical variables selected were compared between TBI participants and HCs at visit 1 to show the differences between the two groups. The Shapiro–Wilk test indicated that the functional (TUG) and biomechanical variables were not normally distributed (*p* < 0.05). The Mann–Whitney *U* test was used to determine the difference between TBI participants and HCs for TUG, gait speed, and biomechanical variables. In addition, Cohen's *d* was computed for all variables.

#### 2.3.3 Regression model

TUG was fit to a linear regression model using the selected visit 1 biomechanical outcomes as independent variables. The developed model was validated using visit 2 TUG measurements. The regression model error with respect to distance was computed and used to conservatively remove gait components from TUG. Specifically, from the TUG value of each participant, we subtracted the time it would take for them to walk 1 m from their independently measured gait speed and fit this value (i.e., TUG—1 m/gait speed) to the same biomechanical variables as the raw TUG model. The TUG test captures the full range of a participant's functional mobility and is not solely focused on balance. So, to understand the effect of gait on TUG, we included gait speed in the model (TUG ∼+GS). However, this model did not provide balance exclusive components. Since the purpose of this manuscript was to understand the balance components measured by TUG, we performed a regression with exclusively balance-focused independent variables that conservatively removed the TUG time associated with gait (TUG-1/GS).

Regression coefficients and their corresponding standard errors are reported, in addition to their level of statistical significance (as indicated in the table footnote). The regression model that best represents TUG was selected.

### 2.4 Sub-population analysis

The Breusch–Pagan test for non-constant variance was used to assess the heteroscedasticity in the regression. Based on the Breusch–Pagan test for non-constant variance, the TBI participants were split into two subgroups. A TUG of 10 s is typically used as a reference point to the upper limit for healthy adults and is associated with increases in fall risk (22, 28, 29). Using a cutoff TUG threshold of 10 s, participants were split into a TUG <10 s group (9 participants) and a TUG ≥10 s group (10 participants). Both TUG and TUG-1/GS models were fit to each subgroup. The TBI population with TUG <10 s was then compared to the HCs using the same models using Cohen's *d*.

All the above models were fit to visit 1 datasets and exclusively trained on visit 1 data. Subsequently, the model's performance was evaluated using visit 2 datasets. This evaluation assessed the robustness of the visit 1-fitted model when applied to previously unobserved data in visit 2 and gauged its generalization performance. The coefficient of determination (*R*^2^) and root-mean-square error (RMSE) were used for model evaluation on both visit 1 and visit 2 datasets. We defined the visit 2 RMSE as the error between the participants on their visit 2 measures and the predictions by the visit 1-fitted model.

## 3 Results

### 3.1 Biomechanical variables

**Figure 2:**
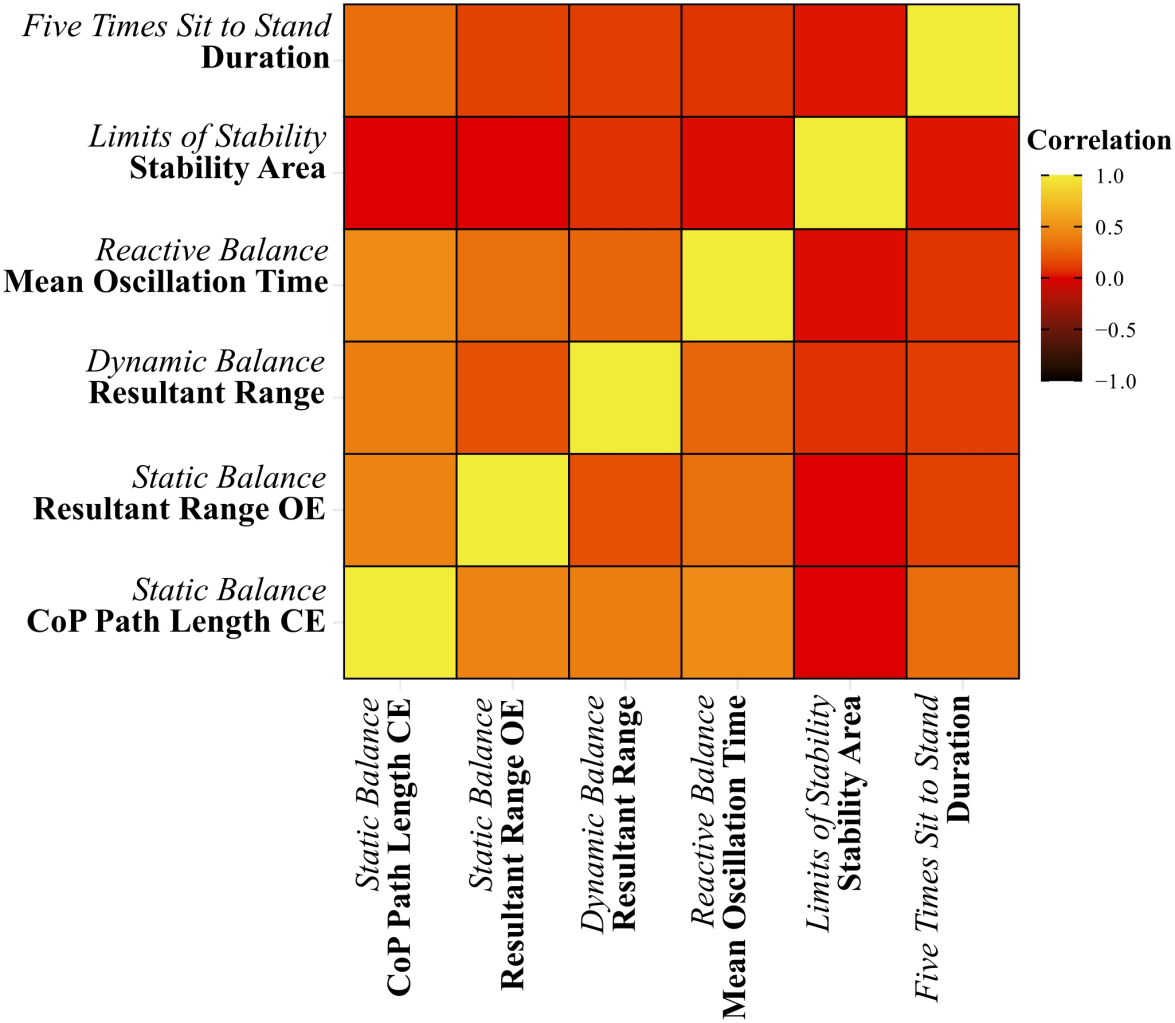
Correlation matrix between selected variables.

Several biomechanical outcomes were collinear, particularly when they were within the same task; hence, one variable was chosen from each task where the co-linearity was the least between the variables across tasks. This resulted in a mean-variance inflation factor of 2.07 (1.27–3.44) among the selected variables (Figure 2). Based on collinearity, the following variables were selected for all models:

(1) Static balance, eyes closed condition—COP path length (Static_EC_COP PL_).
(2) Static balance, EO condition—resultant range trunk oscillations (Static_EO_RR_)
(3) Dynamic balance—resultant range trunk oscillations (Dynamic_RR_)
(4) Reactive balance—mean oscillation time (Reactive_OT_)
(5) Limits of stability—stability area (LOS_SA_)
(6) Sit to stand—duration of the sit-to-stand task (STS_DT_)

### 3.2 Comparison between TBI participants and HCs

Table 2 summarizes the functional measures and balance variables obtained from TBI participants and HCs for visit 1. TBI participants exhibited a significantly longer TUG time (time to complete in seconds) and lower gait speed (m/s) than HC. In addition, both HC and TBI populations shared a significant negative correlation between their TUG and gait speed measurements. For visit 2, the average TUG score of the 15 participants was 11.10 ± 3.49 s.

**Table 2:** Mean and standard deviation for functional and biomechanical outcomes at visit 1.

	TBI	HC	Effect size/ *p*-values
Functional outcomes			
TUG***	11.86 ± 4.35	7.00 ± 1.45	0.70/0.0001
GS***	1.02 ± 0.40	1.80 ± 0.42	0.70/0.0001
Spearman's Rho TUG vs. GS	−0.79	−0.68	
Biomechanical outcomes			
Static_EC_COP PL_	119.17 ± 96.27	65.44 ± 20.18	0.34/0.059
Static_EO_RR_	3.71 ± 1.83	3.31 ± 2.03	0.18/0.325
Dynamic_RR_	12.48 ± 8.50	7.70 ± 4.76	0.35/0.053
Reactive_OT_**	1.63 ± 1.04	0.68 ± 0.22	0.55/0.002
LOS_SA_	94.38 ± 20.92	101.08 ± 18.75	0.21/0.248
STS_DT_*	21.04 ± 6.74	14.75 ± 3.32	0.45/0.011

* *p* < 0.05.

** *p* < 0.01.

*** *p* < 0.001.

The biomechanical outcomes (reactive oscillation time and duration of the sit-to-stand task) also showed a significant difference between HC and TBI participants (Table 2). The TBI participants took about 1 s longer to stabilize their trunk after a platform perturbation than HCs, as observed by the mean reactive oscillation time. TBI participants also took an average of 5 s longer to complete the five times sit-to-stand exercise than the HCs. Although not significant, the mean COP PL during the quiet standing with EC task and the trunk oscillation range during the dynamic balance task were almost twice in TBI participants compared to HCs, and the stability area in the limits of stability task was also greater in HCs than participants with TBI.

### 3.3 Regression model

The TUG model is shown as follows:

(4) $$\mathrm{TUG}=\mathrm{Static}\_EC_{\mathrm{COP}\;\mathrm{PL}}+\mathrm{Static}\_EO_{\mathrm{RR}}+\mathrm{Dynami}c_{\mathrm{RR}}+\mathrm{Reactiv}e_{\mathrm{OT}}+\mathrm{LO}S_{\mathrm{SA}}+\mathrm{ST}S_{\mathrm{DT}}+\mathrm{intercept}$$

**Figure 3:**
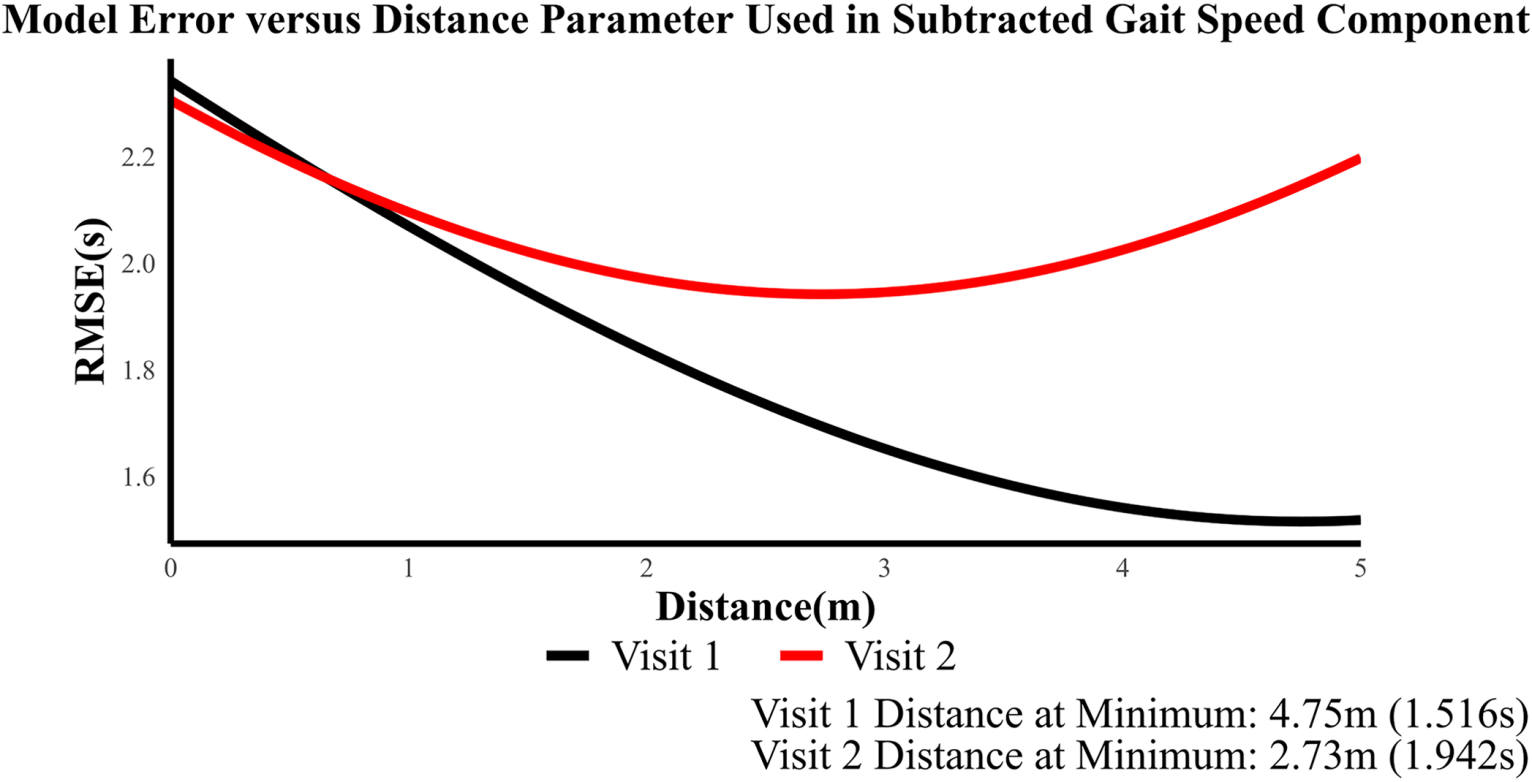
Distance in gait speed vs. model's RMSE.

The performance and parameter estimate of each regression model applied to the TBI and HC populations is presented in Table 3. The gait speed component was subtracted conservatively from TUG to remove gait components. Figure 3 shows the model error with respect to the distance in gait speed parameter. The results showed that at ∼2 m model error was minimum. So, to conservatively remove the gait from TUG, we removed time to walk 1 m. We hypothesize that TUG scores will be related to dynamic and reactive balance variables.

**Table 3:** Regression coefficients and standard errors for the TUG, TUG ∼ + GS, and TUG-1/GS models.

	TBI			HC	
	TUG	TUG-1/GS	TUG ∼ + GS	TUG	TUG-1/GS
*R* ^2^	0.695	0.685	0.789	0.860	0.883
Visit 1/visit 2 RMSE	2.342/2.306	2.070/2.096	1.948/1.985	0.518/—	0.431/—
(Intercept)	4.17 (4.58)	4.78 (4.05)	14.27 (6.05)*	10.36 (2.90)*	9.35 (2.41)*
Static_EC_COP PL_	0.00 (0.01)	0.00 (0.01)	0.00 (0.01)	−0.03 (0.02)	−0.03 (0.02)
Static_EO_RR_	−0.14 (0.52)	−0.14 (0.46)	−0.15 (0.45)	−0.09 (0.14)	−0.06 (0.11)
Dynamic_RR_	−0.25 (0.12)	−0.20 (0.10)	−0.16 (0.10)	−0.09 (0.10)	−0.07 (0.08)
Reactive_OT_	**3.23** (0.99)**	**2.75** (0.88)**	1.95 (1.04)	6.08 (2.56)	**5.57** (2.13)*
LOS_SA_	0.00 (0.04)	−0.01 (0.03)	−0.00 (0.03)	**−0.06** (0.02)*	**−0.05** (0.01)*
STS_DT_	0.25 (0.13)	0.22 (0.11)	0.10 (0.13)	0.12 (0.08)	0.10 (0.07)
GS	—	—	**−5.36** (2.42)*	—	—

The bold values are statistically significant non-intercept effects.

* *p* < 0.05.

** *p* < 0.01.

**Figure 4:**
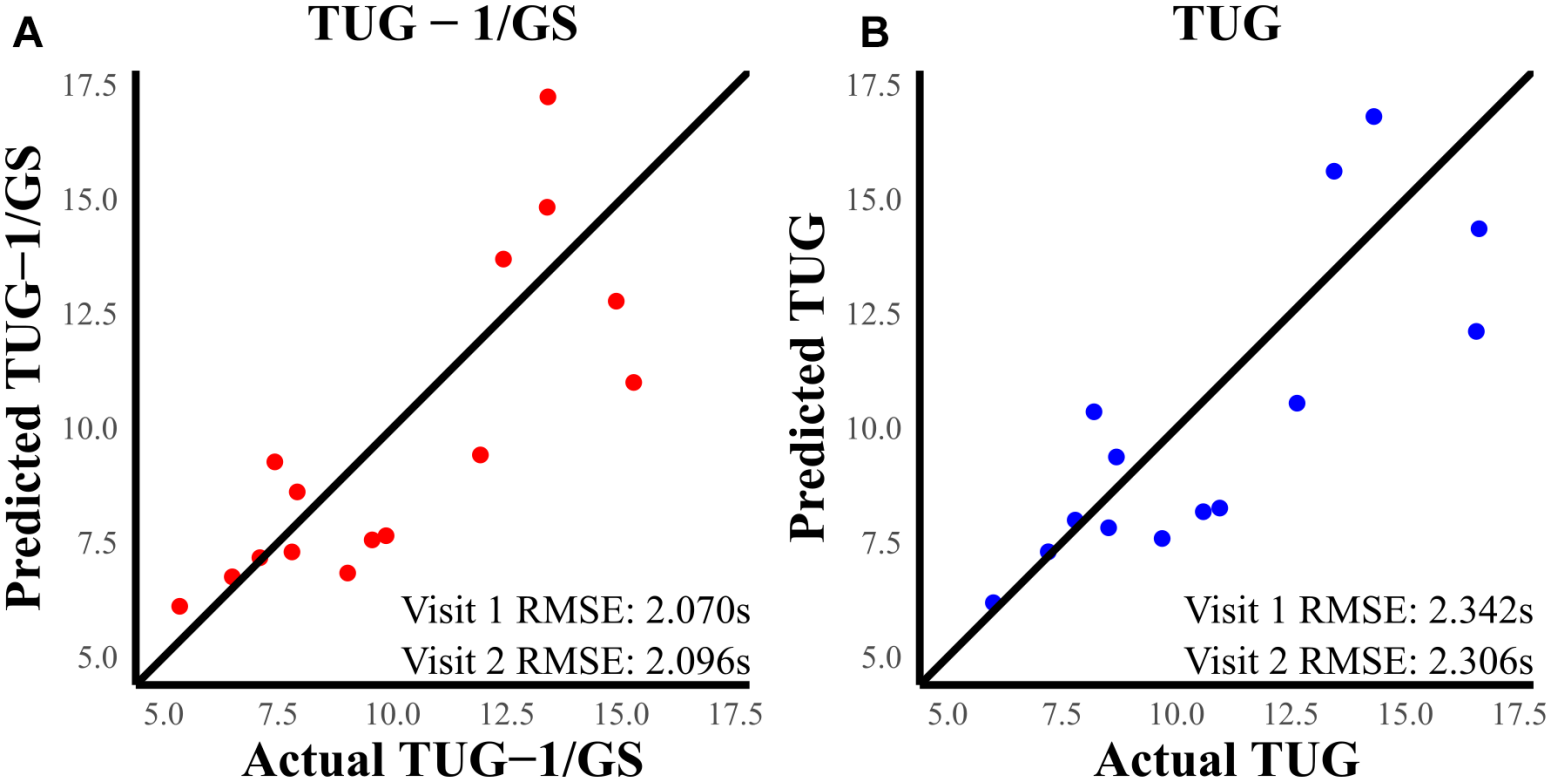
(**A**) Predicted TUG-1/GS vs. actual TUG-1/GS at visit 2. (**B**) Predicted TUG vs. actual TUG at visit 2. The RMSE was better in the TUG-1/GS model compared to TUG.

RMSE (Figure 4) shows the TBI models [TUG (Equation 4) and TUG-1/GS (Equation 6)]; Figure 4 explains over 68% of the variability in their respective outcomes and shows a consistent RMSE between visit 1 and visit 2 datasets. The TUG-1/GS TBI model achieved the best RMSE at nearly ±2 s during both visit 1 and follow-up (Table 3). HC models explained over 86% of the TUG variability and captured TUG to ±0.5 s (Table 3). Regression analysis revealed a significant positive association between TUG performance and mean reactive oscillation time in TBI models (TUG and TUG-1/GS) and the HC model (TUG-1/GS). The TUG ∼ + GS (Equation 5) model was also assessed in this case where only gait speed showed a significant effect.

The TUG ∼ + GS model is given as follows:

(5) $$\mathrm{TUG}=\mathrm{Static}\_EC_{\mathrm{COP}\;\mathrm{PL}}+\mathrm{Static}\_EO_{\mathrm{RR}}+\mathrm{Dynami}c_{\mathrm{RR}}+\mathrm{Reactiv}e_{\mathrm{OT}}+\mathrm{LO}S_{\mathrm{SA}}+\mathrm{ST}S_{\mathrm{DT}}+\mathrm{GS}+\mathrm{intercept}$$

The TUG-1/GS model is given as follows:

(6) $$\mathrm{TUG}\text{-}1/\mathrm{GS}=\mathrm{Static}\_EC_{\mathrm{COP}\;\mathrm{PL}}+\mathrm{Static}\_EO_{\mathrm{RR}}+\mathrm{Dynami}c_{\mathrm{RR}}+\mathrm{Reactiv}e_{\mathrm{OT}}+\mathrm{LO}S_{\mathrm{SA}}+\mathrm{ST}S_{\mathrm{DT}}+\mathrm{intercept}$$

#### 3.3.1 Sub-population analysis

**Figure 5:**
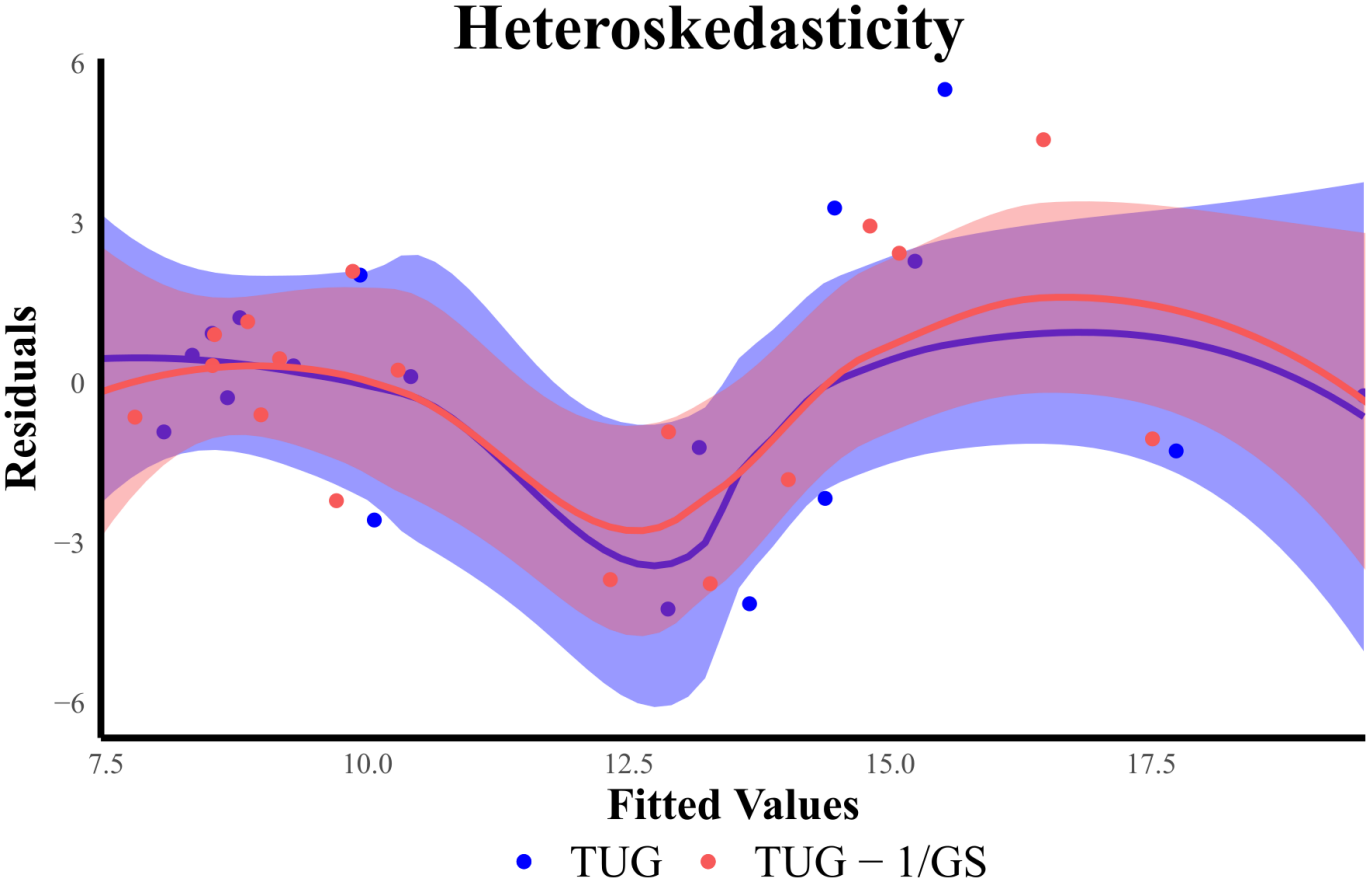
Heteroskedasticity shown using residuals.

Heteroskedasticity was assessed for TUG and TUG-1/GS models because these two models showed the best results. There was an increase in the spread of TUG predictions as the actual TUG measurements increased (Figure 5). Although a test for non-constant error was not significant (*p* = 0.103), the phenomenon can be visually observed in both visit 1 and visit 2 (Figure 5) datasets. In the TUG-1/GS model, this heteroskedasticity is only marginally reduced (*p* = 0.123). This same phenomenon was not observed in the healthy dataset. To reduce this heteroskedasticity, the data were split into two sub-populations (TUG <10 s and TUG ≥10 s) based on TUG.

Table 4 shows the functional measure statistics after splitting TBI into groups based on a threshold TUG of 10 s. A large significant change in TUG variability was observed between splits (*F*-statistic 17.525, *p* < 0.001), whereas this change was not observed with gait speed (*F*-statistic 0.94472, *p* > 0.1).

**Table 4:** Mean and standard deviation for functional and biomechanical outcomes at visit 1 and visit 2.

	Visit 1			Visit 2	
	TUG ≥10 (*n* = 10)	TUG <10 (*n* = 9)	Effect size: TBI <10 vs. HC)	TUG ≥10 (*n* = 8)	TUG <10 (*n* = 7)
Functional outcomes					
TUG	14.88 ± 3.97	8.51 ± 0.95		13.79 ± 2.32	8.01 ± 1.18
GS	0.80 ± 0.32	1.27 ± 0.33		0.91 ± 0.28	1.42 ± 0.09
Biomechanical outcomes					
Static_EC_COP PL_	171.53 ± 107.72	61.00 ± 23.84	0.16	175.12 ± 165.74	69.33 ± 41.69
Static_EO_RR_	4.43 ± 1.75	2.91 ± 1.66	0.02	6.40 ± 2.09	2.49 ± 0.8
Dynamic_RR_	15.03 ± 10.33	9.65 ± 5.04	0.19	13.26 ± 9.69	8.13 ± 4.33
Reactive_OT_	2.23 ± 1.07	0.96 ± 0.45	0.29	1.80 ± 1.36	0.68 ± 0.20
LOS_SA_	90.70 ± 23.78	98.47 ± 17.67	0.16	88.50 ± 10.15	96.49 ± 19.48
STS_DT_	23.42 ± 7.44	18.40 ± 4.98	0.34	25.10 ± 7.63	14.19 ± 1.53

**Figure 6:**
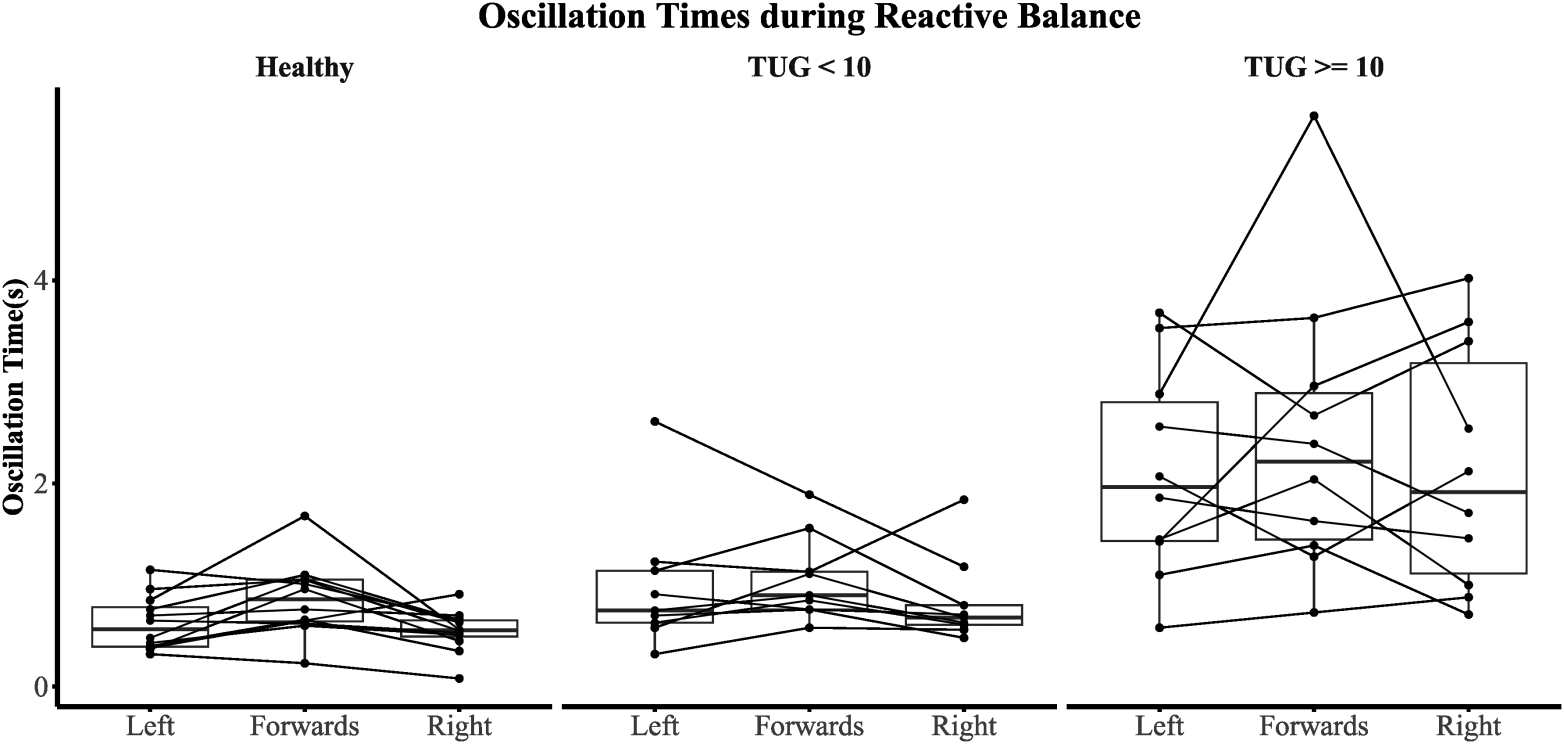
Oscillation time across all perturbation types. A participant is represented by a single line.

The sub-population regression models are presented in Table 5. *R*^2^ was greater than 0.83 for all sub-population models. The TUG <10 s models achieved smaller visit 1 and visit 2 RMSEs than TUG ≥10 s models. However, the visit 2 RMSE was exceptionally poor in the TUG ≥10 s models, with an average follow-up RMSE of ±4 s. In the TUG ≥10 s population models, the effects of reactive balance oscillation time mean and dynamic balance resultant range were statistically significant (both models, *p* < 0.05). However, for the TUG <10 s models, the effect of reactive balance was diminished. As depicted in Figure 6, the individual contributing components to the reactive balance oscillation time mean reveal similar tendencies within both the HC and TBI populations with TUG scores <10 s. Notably, the effect size (Cohen's *d*) between the TBI group with TUG scores <10 s and the HC group was 0.29, indicating a moderate difference. These results suggest that variations in dynamic and reactive balance are associated with variations in TUG when TUG is ≥10 s, while reactive balance is associated with variations in TUG when TUG is <10 s. This suggests that TUG could inform clinicians about dynamic and reactive balance components and thus could guide therapy progression.

**Table 5:** Regression coefficients and standard errors for the TUG and TUG-1/GS models for the TBI sub-population.

	TUG		TUG-1/GS	
	TUG <10	TUG ≥10	TUG <10	TUG ≥10
*R* ^2^	0.836/0.342	0.904/0.712	0.866/0.463	0.878/0.633
Visit 1/visit 2 RMSE	0.363/1.456	1.167/4.398	0.310/1.512	1.099/3.565
(Intercept)	2.58 (3.59)	0.12 (4.68)	1.10 (3.07)	2.40 (4.40)
Static_EC_COP PL_	−0.01 (0.02)	0.00 (0.01)	−0.01 (0.01)	0.00 (0.01)
Static_EO_RR_	0.29 (0.20)	−0.73 (0.65)	0.28 (0.17)	−0.70 (0.61)
Dynamic_RR_	0.08 (0.07)	−0.49 (0.12)*	0.10 (0.06)	−0.39 (0.12)*
Reactive_OT_	1.25 (1.04)	4.15 (0.97)*	1.42 (0.89)	3.37 (0.91)*
LOS_SA_	0.01 (0.02)	0.08 (0.04)	0.02 (0.02)	0.06 (0.04)
STS_DT_	0.15 (0.06)	0.34 (0.13)	0.13 (0.05)	0.28 (0.12)

* *p* < 0.05.

## 4 Discussion

TBI is one of the leading causes of balance deficits in adults and leads to reduced activities of daily living and increased fall risk (1, 2). The TUG test is one of the most widely used clinical measures to assess the gait, balance, and fall risk in individuals with TBI (7–10). Although TUG has been used extensively, limited research has been published that investigates the relationship between TUG performance and quantitative biomechanical measures of balance. This study presents the key findings to understand the relationship between biomechanical variables of balance and TUG performance, for both healthy individuals and participants with TBI. Using a linear model, we demonstrated this relationship and extended our analysis to include the visit 2 dataset to explore the model's generalization ability. Furthermore, within the dataset with TBI participants, we conducted a deeper exploration by dividing it into subgroups based on lower and higher TUG scores to provide preliminary evidence of variable changes after such a split. This investigation revealed that reactive balance and dynamic balance emerged as prominent features that delineate the relationship between balance parameters and TUG scores within this subgroup. These findings offer valuable insights into the nuanced nature of this relationship and its potential implications for assessment and intervention strategies in individuals with TBI.

Biomechanical variables provide quantitative insights into the underlying impairment in balance mechanisms and their recovery. Understanding the changes in these mechanisms will help us understand the reasons for the observed functional changes and recovery. Thus, understanding the relationship between TUG performance and biomechanical metrics will help comprehend the reason for the functional deficit, which will assist in the development of personalized interventions for individuals with TBI based on their deficits and may ultimately improve TBI rehabilitation.

Nineteen participants diagnosed with TBI and 12 HCs participated in this study. TBI participants showed significantly reduced gait and balance function (as measured by the TUG score and gait speed) and decreased biomechanical outcomes (oscillation time during reactive balance tasks and time duration to perform the sit-to-stand task) compared to HCs, as shown in Table 2. COP path length during quiet standing with eyes closed and the resultant range during the dynamic balance test also showed differences between TBI participants and HCs, as observed by their high effect sizes. A regression model was constructed using these six biomechanical variables, and it was able to predict TUG and TUG-1/GS scores at about ±2 s for both visit 1 and visit 2 datasets. The model with conservatively removed gait speed (i.e., TUG-1/GS) gave the best results, maintaining a consistent RMSE at ∼ ± 2 s at both visit 1 and visit 2 and explaining over 68% of the variability. Since the TUG measure captures the full range of a participant's functional mobility and is not solely focused on balance (30), we wanted to remove the effects of gait conservatively. Gait speed, as a measure of functional ambulation, is also associated with dynamic balance and is strongly correlated to TUG. Thus, removing the contribution of gait, which overlaps with TUG, is difficult. This effect is clear when we include gait speed as a variable in the regression model (TUG ∼+GS), which removed the significance of trunk stability during the reactive balance task. Hence, to isolate the balance component, 1 m/gait speed was subtracted from the time. Subtracting the 1 m/gait speed (i.e., time to walk 1 m) from the overall TUG time has the advantage of minimizing error in the observed data since it effectively reduces the spread of TUG values. When the distance (i.e., numerator) was increased, the error was minimized, with the effect reaching a plateau, as shown in Figure 3. We conservatively chose a value of 1  m/gait speed to subtract from TUG [as opposed to the value that minimizes error (from Figure 3), as it is not guaranteed that increasing the numerator will yield better predictive results] to not remove any balance components from unobserved datasets.

The regression model showed that oscillation time during the reactive balance task had a significant association with TUG. Similar effects were also observed in the HC TUG-1/GS model. Oscillation time was significantly longer in TBI participants than in HCs (Table 3). Research has shown that TBI can impair sensory (visual, vestibular, and proprioceptive) pathways or their integration that permits body position awareness and efficient reaction to internal (i.e., hand swing) and external (i.e., environmental) perturbations (6). These sensory deficits combined with attention deficits in TBI can result in decreased postural responses to perturbation, resulting in large trunk oscillations with prolonged time to converge to stability, ultimately leading to decreased balance and increased fall risk. These results confirm that the TUG test is an excellent predictor of fall risk, as observed in previous research (31, 32).

The TUG-1/GS model presented in this study could explain over 69% of the response variability for TBI participants and 88% for HC. The minimum clinical important difference (MCID) for TBI was 1.6 s in TUG (33); therefore, TUG models with an RMSE value of ∼2 s were considered relatively imprecise. However, the sub-population models TUG <10 s and TUG **≥**10 s explained 86% and 88% of the response variability, respectively, and increased precision to <1.2 s RMSE.

In the sub-population analysis, the TUG >10 s cohort showed a significant relationship between the TUG score and oscillation time during the reactive balance task and the resultant range during the dynamic balance task. Compared to HCs, the TBI participants also showed a large resultant range during the dynamic balance task and a longer oscillation time during the reactive balance task. Dynamic balance was used to quantify the participants’ ability to maintain an upright posture without any information from the proprioceptive system (i.e., information from participants’ ankles and feet). The participants relied on information from visual and the vestibular system to overcome the lack of reliable sensory or proprioceptive information from their ankles and feet. This suggests that TBI participants have increased reliance on sensory systems for balance. The results suggest that at higher TUG scores, the effect of this sensory reliance on functional balance is more evident. The TUG <10 s cohort did not exhibit a significant relationship between the TUG score and reactive balance, unlike the observed trend in the model with the entire TBI cohort or the healthy cohort, despite having a high regression coefficient. This could be due to TUG not being sensitive at lower values or the smaller sample size of the cohort. Although the TUG-1/GS and TUG models showed better results in the sub-population at visit 1, their performance deteriorated at visit 2, which could be due to the low sample size at follow-up.

Although the static balance during closed eyes condition exhibited a high Cohen's *d* (Table 2) for TBI participants compared to HCs, it did not show any association with TUG. This could be due to the sensitivity of TUG (Table 3) to visual feedback or other metrics removing the significance of this metric.

The current results show that TUG is highly associated with reactive balance and is a good predictor of postural stability after perturbations. At higher TUG values, TUG may also differentiate the effects of sensory deficits on balance. Although the current model provides valuable insight into TUG, the model is limited by the slightly high observed RMSE, which could be due to the small sample size with high variability. For the purpose of this paper, we also did not employ any feature selection strategy to optimize our model, opting instead for the manual selection of variables. These are avenues to explore in future work.

### 4.1 Limitations of the study

The study is limited by the smaller sample size and the smaller number of participants in each subgroup analysis. Although we validated our results using another timepoint visit 2, future studies need to further evaluate this model using a larger sample size. In addition, the sample needs to be expanded to all age groups, and analysis should be performed with age as a covariate to understand the effects of age on the parameters.

### 4.2 Clinical implications of study

Balance deficits could be due to the deterioration of various components of the neuromuscular system, such as sensory integration and the generation of appropriate muscular responses. These deteriorations could be quantitatively evaluated using biomechanical metrics. Clinical assessments like as the TUG test provide a reliable and cost-effective measure to evaluate balance deficits. Understanding the relationship between functional and biomechanical metrics could help understand the underlying mechanism responsible for functional deficits. This could help provide personalized, targeted interventions based on individuals’ residual motor and balance deficits.

## Data Availability

The datasets presented in this article are not readily available because the current IRB does not allow data sharing. Requests to access the datasets should be directed to kkarunakaran@kesslerfoundation.org.

## References

[1] Singh J. Olabode reference centers for disease control and prevention. Indian J Pharmacol. (2004) 36:268–9.

[2] NJ Department of Health. Available online at: https://nj.gov/health/ (accessed May 9, 2023).

[3] Newton RA. Balance abilities in individuals with moderate and severe traumatic brain injury. Brain Inj. (1995) 9:445–51. 10.3109/02699059509008204 7550216

[4] Williams G Morris ME Schache A McCrory PR. Incidence of gait abnormalities after traumatic brain injury. Arch Phys Med Rehabil. (2009) 90:87–593. 10.1016/j.apmr.2008.10.013 19345773

[5] Santos MJ Kanekar N Aruin AS. The role of anticipatory postural adjustments in compensatory control of posture: 2. Biomechanical analysis. J Electromyogr Kinesiol. (2010) 20:398–405. 10.1016/j.jelekin.2010.01.002 20156693 PMC2859839

[6] Aruin AS. Enhancing anticipatory postural adjustments: a novel approach to balance rehabilitation. J Nov Physiother. (2016) 6(2):e144. 10.4172/2165-7025.1000e144 27335705 PMC4913780

[7] Katz-Leurer M Rotem H Lewitus H Keren O Meyer S. Functional balance tests for children with traumatic brain injury: within-session reliability. Pediatr Phys Ther. (2008) 20:254–8. 10.1097/PEP.0b013e3181820dd8 18703963

[8] Shukla D Devi BI Agrawal A. Outcome measures for traumatic brain injury. Clin Neurol Neurosurg. (2011) 113:435–41. 10.1016/j.clineuro.2011.02.013 21440363

[9] Graff K Szczerbik E Kalinowska M Kaczmarczyk K Stepien A Syczewska M. Using the TUG test for the functional assessment of patients with selected disorders. Int J Environ Res Public Health. (2022) 19. 10.3390/ijerph19084602 PMC9025107 35457472

[10] Nightingale CJ Mitchell SN Butterfield SA. Validation of the Timed Up and Go test for assessing balance variables in adults aged 65 and older. J Aging Phys Act. (2019) 27:230–3. 10.1123/japa.2018-0049 30117359

[11] Beerse M Lelko M Wu J. Biomechanical analysis of the Timed Up-and-Go (TUG) test in children with and without Down syndrome. Gait Posture. (2019) 68:409–14. 10.1016/j.gaitpost.2018.12.027 30594868

[12] Adachi D Nishiguchi S Fukutani N Hotta T Tashiro Y Morino S Generating linear regression model to predict motor functions by use of laser range finder during TUG. J Orthop Sci. (2017) 22:549–53. 10.1016/j.jos.2017.01.020 28254157

[13] Winter DA. Human balance and posture control during standing and walking. Gait Posture. (1995) 3:193–214. 10.1016/0966-6362(96)82849-9

[14] DeBerardinis J Neilsen C Lidstone DE Dufek JS Trabia MB. A comparison of two techniques for center of pressure measurements. J Rehabil Assist Technol Eng. (2020) 7:2055668320921063. 10.1177/2055668320921063 32670601 PMC7338728

[15] Jamshidi N Rostami M Najarian S Menhaj MB Saadatnia M Salami F. Differences in center of pressure trajectory between normal and steppage gait. J Res Med Sci. (2010) 15(1):33–40. . 21526056 PMC3082780

[16] Chiari L Dozza M Cappello A Horak FB Macellari V Giansanti D. Audio-biofeedback for balance improvement: an accelerometry-based system. IEEE Trans Biomed Eng. (2005) 52:2108–11. 10.1109/TBME.2005.857673 16366234

[17] Heebner NR Akins JS Lephart SM Sell TC. Reliability and validity of an accelerometry based measure of static and dynamic postural stability in healthy and active individuals. Gait Posture. (2015) 41:535–9. 10.1016/j.gaitpost.2014.12.009 25544692

[18] Marchesi G Casadio M Ballardini G Luca A Squeri V Vallone F Robot-based assessment of sitting and standing balance: preliminary results in Parkinson’s disease. IEEE Int Conf Rehabil Robot. (2019) 2019:570–6. 10.1109/ICORR.2019.8779387 31374691

[19] Marchesi G Ricaldone E De Luca A Torre K Quinland E A robot-based assessment of trunk control in Spinal Cord Injured athletes. In 2020 8th IEEE RAS/EMBS International Conference for Biomedical Robotics and Biomechatronics (BioRob). New York: IEEE (2020). p. 497–502. 10.1109/BioRob49111.2020.9224337

[20] Reynard F Christe D Terrier P. Postural control in healthy adults: determinants of trunk sway assessed with a chest-worn accelerometer in 12 quiet standing tasks. PLoS ONE. (2019) 14(1):e0211051. 10.1371/journal.pone.0211051 30673753 PMC6344019

[21] Row J Chan L Damiano D Shenouda C Collins J Zampieri C. Balance assessment in traumatic brain injury: a comparison of the sensory organization and limits of stability tests. J Neurotrauma. (2019) 36:2435. 10.1089/neu.2018.5755 30909842 PMC6661911

[22] SRA Lab. *Rehab measures*. Available online at: https://www.sralab.org/rehabilitation-measures. (accessed May 9, 2023).

[23] Saglia JA De Luca A Squeri V Ciaccia L Sanfilippo C Ungaro S Design and development of a novel core, balance and lower limb rehabilitation robot: Hunova®. In 2019 IEEE 16th International Conference on Rehabilitation Robotics (ICORR), Toronto, ON, Canada. (2019). p. 417–22. 10.1109/ICORR.2019.8779531 31374665

[24] Pilkar R Veerubhotla A Ehrenberg N. Objective evaluation of the risk of falls in individuals with traumatic brain injury: feasibility and preliminary validation. In 2021 43rd Annual International Conference of the IEEE Engineering in Medicine & Biology Society (EMBC), Mexico. (2021). p. 4658–61. 10.1109/EMBC46164.2021.9630020 34892252

[25] Marchesi G De Luca A Squeri V De Michieli L Vallone F Pilotto A A lifespan approach to balance in static and dynamic conditions: the effect of age on balance abilities. Front Neurol. (2022) 13:29. 10.3389/fneur.2022.801142 PMC8899125 35265025

[26] Giovannini S Iacovelli C Brau F Loreti C Fusco A Caliandro P Robotic-assisted rehabilitation for balance and gait in stroke patients (ROAR-S): study protocol for a preliminary randomized controlled trial. Trials. (2022) 23:1–10. 10.1186/s13063-022-06812-w 36224575 PMC9558956

[27] De Luca A Squeri V Barone LM Vernetti Mansin H Ricci S Pisu I Dynamic stability and trunk control improvements following robotic balance and core stability training in chronic stroke survivors: a pilot study. Front Neurol. (2020) 11:494. 10.3389/fneur.2020.00494 32625162 PMC7311757

[28] Bohannon RW. Reference values for the timed up and go test: a descriptive meta-analysis. J Geriatr Phys Ther. (2006) 29(2):64–8. 10.1519/00139143-200608000-00004 16914068

[29] Kear BM Guck TP McGaha AL. Timed up and go (TUG) test: normative reference values for ages 20 to 59 years and relationships with physical and mental health risk factors. J Prim Care Community Health. (2017) 8(1):9–13. 10.1177/2150131916659282 27450179 PMC5932649

[30] Herman T Giladi N Hausdorff JM. Properties of the “Timed Up and Go” test: more than meets the eye. Gerontology. (2011) 57:203. 10.1159/000314963 20484884 PMC3094679

[31] Barry E Galvin R Keogh C Horgan F Fahey T. Is the timed up and go test a useful predictor of risk of falls in community dwelling older adults: a systematic review and meta- analysis. BMC Geriatr. (2014) 14:14. 10.1186/1471-2318-14-14 24484314 PMC3924230

[32] Klima D Morgan L Baylor M Reilly C Gladmon D Davey A. Physical performance and fall risk in persons with traumatic brain injury. Percept Mot Skills. (2019) 126:50. 10.1177/0031512518809203 30458668 PMC7286440

[33] Katz-Leurer M Rotem H Keren O Meyer S. The effects of a ‘home-based’ task-oriented exercise programme on motor and balance performance in children with spastic cerebral palsy and severe traumatic brain injury. Clin Rehabil. (2009) 23(8):714–24. 10.1177/0269215509335293 19506005

